# 

**Published:** 1903-11

**Authors:** 


### CONTENTS.

ORIGINAL COMMUNICATIONS—

A PLEA FOR THE MICROSCOPIC EXAMINATION OF THE BLOOD IN THE CONTINUED AND REMITTENT FEVERS COMMON TO OUR SOUTHERN STATES. By Louis M. Warfield, A.B., M.D., Savannah, Ga.... 505

SOME REMARKS ON DIAGNOSIS AND TREATMENT OF VENEREAL ULCERS. By W. B. Emery, M.D., Atlanta, Ga.... 510

REPORT OF ONE HUNDRED AND FIFTY CASES RECEIVING THE PASTEUR TREATMENT AT THE GEORGIA PASTEUR INSTITUTE By James N. Brawner, M.D., Atlanta, Ga.... 516

EDITORIAL NOTES AND COMMENTS—

THE TRI-STATE MEDICAL SOCIETY.... 519

A MESSIANIC TRINITY.... 523

NEWS AND NOTES.... 526

CONTENTS CONTINUED.... X

